# Influence of Functional Knee Bracing on the Isokinetic and Functional Tests of Anterior Cruciate Ligament Deficient Patients

**DOI:** 10.1371/journal.pone.0064308

**Published:** 2013-05-23

**Authors:** Niyousha Mortaza, Noor Azuan Abu Osman, Ali Ashraf Jamshidi, Javad Razjouyan

**Affiliations:** 1 Department of Biomedical Engineering, Faculty of Engineering, University of Malaya, Kuala Lumpur, Malaysia; 2 Department of Orthotics and Prosthetics, Faculty of Rehabilitation, Tehran University of Medical Sciences, Tehran, Iran; 3 Department of Physical Therapy, Faculty of Rehabilitation, Tehran University of Medical Sciences, Tehran, Iran; 4 Rehabilitation Research Centre, Faculty of Rehabilitation, Tehran University of Medical Sciences Tehran, Iran; 5 Department of Electrical and Electronics Engineering, Garmsar Branch, Islamic Azad University, Garmsar, Iran; Universidad Europea de Madrid, Spain

## Abstract

Use of functional knee braces has been suggested to provide protection and to improve kinetic performance of the knee in Anterior cruciate ligament(ACL)-injured patients. However, many athletes might refrain from wearing the braces because of the fear of performance hindrance in the playing field. The aim of this study was to examine the effect of three functional knee brace/sleeves upon the isokinetic and functional performance of ACL-deficient and healthy subjects. Six anterior cruciate ligament deficient (29.0±5.3 yrs., 175.2±5.4 cm, and 73.0±10.0 kg) and six healthy male subjects (27.2±3.7 yrs., 176.4±6.4 cm, and 70.3±6.9 kg) were selected. The effect of a custom-made functional knee brace, and two neoprene knee sleeves, one with four metal supports and one without support were examined via the use of isokinetic and functional tests in four sets (non-braced,wearing functional knee brace,and wearing the sleeves). Cross-over hop and single leg vertical jump test were performed and jump height, and hop distance were recorded. Peak torque to body weight ratio and average power in two isokinetic velocities(60°.s^−1^,180°.s^−1^) were recorded and the brace/sleeves effect was calculated as the changes in peak torque measured in the brace/sleeves conditions, expressed as a percentage of peak torque measured in non-braced condition. Frequency content of the isokinetic torque-time curves was also analyzed. Wilcoxon signed rank test was used to compare the measured values in four test conditions within each control and ACL-deficient group,and Mann-Whitney U test was used for the comparison between the two groups. No significant differences in peak torque, average power, torque-time curve frequency content, vertical-jump and hop measurements were found within the experimental and the non-braced conditions (p>0.05). Although the examined functional knee brace/sleeves had no significant effect on the knee muscle performance, there have been some enhancement regarding the extension peak torques and power generating capacity of the ACL-deficient subjects that could be helpful in reducing the bilateral asymmetry in these patients.

## Introduction

Use of functional knee braces (FKB) has been suggested to provide protection and to improve kinetic performance of the knee in ACL-injured patients [1]–[3]. However, the efficacy of knee bracing in achieving these goals is still controversial [4], [5]. On the other hand, the results of the subjective evaluations revealed that patients responded positively to wearing the braces indicating better knee stability, performance and pain alleviation [4], [6]. Despite this many athletes still refrain from wearing the braces because of the fear of performance hindrance in the playing field [7], [8]. Hence, further studies seem to be necessary in order to detect the effects of knee braces on the performance.

The results of isokinetic tests on the immediate effect of knee braces showed that, depending on the patient's strength, injury type, previous history of wearing the knee braces and brace design, the protective knee braces can have some deteriorating effect on the torque generating capacity of the knee musculature [3], [9]–[12]. In all of these studies the conventional isokinetic results such as peak torque to body weight ratio (PTBWR), average power and total work were considered. However, these assessments are not accurate enough to evaluate the quality of torque generation throughout the range of flexion and extension. The oscillation patterns of torque time curves could be indicative of the force generation quality of the joint muscles. Consequently, applying a method to quantify these oscillations in the isokinetic torque pattern could give a more accurate result in assessing the effect of different sorts of treatment including brace application [13], [14].

The purpose of the present study was: first, to find out if the functional knee braces are beneficial in enhancing the force control capacity of the anterior cruciate ligament-deficient (ACLD) patients Through frequency domain analysis, second, to examine the effects of a functional knee brace and two neoprene knee sleeves on the isokinetic and functional tests in patients with ACLD, and last, to find out if there is any relationship between the effect of the brace on the isokinetic muscle performance and the subjects' muscular strength, and if the brace/sleeve had different effects on healthy subjects. The results of this study will help the rehabilitation team to decide on the knee brace/sleeve use after the acute phase of ACL injury, considering the patients' functional performance and current strength status.

## Methods

### Participants

This study was conducted on six male subjects with ACLD (29.0±5.3 yrs., 175.2±5.4 cm, and 73.0±10.0 kg) and 6 healthy subjects as the healthy control group. The control group were healthy subjects matched for height, age and weight (27.2±3.7, 176.4±6.4 cm, 70.3±6.9) to the ACLD group. The time lapse since the episode of injury for the ACLD group was 3.0±1.1 months. The diagnosis of ACL injury was established by physical examination and MRI. The inclusion criteria for the patient group included: (1) grade 3 of muscle strength in quadriceps and hamstring muscles based on manual muscle test, (2) full extension range on the affected side, (3) completed physical therapy rehabilitation period. All the ACLD subjects have completed the same physical therapy rehabilitation treatment which included ten sessions of perturbation training (in the category of neuromuscular training). The healthy group had no history of knee injury and no evidence of any knee instability.

### Ethics Statement

Approval was received from the Ethics Committee of Tehran University of Medical Sciences. Prior to the start of the study, informed written consent was obtained from each subject.

### FKB/Neoprene Sleeves

**Figure 1:**
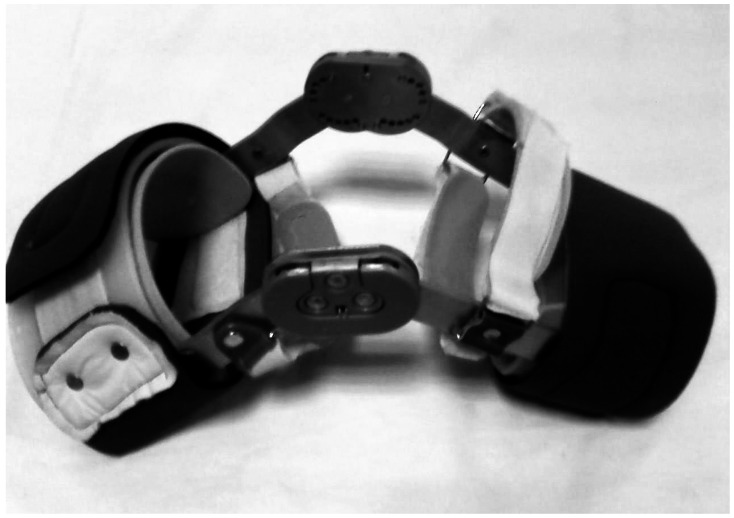
Functional knee brace.

Two neoprene knee sleeves and one custom-made FKB were used. One of the sleeves had four metal supports on each side and the other one was with the exact design but without the metal supports. The FKB consisted of bilateral aluminium bars with polycentric knee joints, two plastic posterior thigh and cuff shells, and Velcro attached to neoprene bands as closures (Figure 1).The braces were fabricated and fitted by a certified orthotist. The custom-made FKB is designed to restrict the anterior translation of tibia relative to femur; the bilateral hinges allowed full extension and flexion of the knee joint.

### Experimental design

All tests were performed unilaterally on the ACLD side. For each subject, the tests were performed identically in four conditions: (1) non-braced (NBR) or control (2) with simple neoprene knee sleeve (3) with neoprene sleeve with four metal supports (4) with Custom-made FKB. The test order was randomized. Subjects were allowed a minimum of 10- minutes of rest between tests. Prior to the start of testing, all subject performed pre-established warm up on a stationary bicycle for 5–8 minutes followed by 3–5 minutes of whole body stretching.

#### Functional tests

In this study two different functional tests including crossover hop for distance and single leg vertical jump (SLVJ) test were included to measure the possible effect of FKB/sleeves on the knee performance. Subjects fulfilled up to three practice tests. Then, they first performed the cross hop test for distance by performing four consecutive hops, crossing the centre line with each hop, more details are presented in the previous study [12]. Next, SLVJ test began with a measure of reach height on the dominant side next to the wall with ink applied to the participants' finger. Then, they performed a maximal effort single-leg jump and reached to touch the wall placing the second mark at the pick of the jump. Participants were allowed to land bilaterally, as this is a more functional movement pattern [15]. In both tests, no restrictions were placed on the upper extremity movements. Each subject was given three trials, and the average jump distance of the three trials was calculated.

#### Isokinetic tests

Isokinetic measurements were performed after functional tests, using Biodex Multi-Joint System 3 (Biodex Medical Systems, Inc., New York, USA) dynamometer with the knee attachment on. Participants were seated and secured to the apparatus with straps across the chest and thighs. The back of the seat was set at an 85° angle. The resistance pad was placed on the distal tibia [12]. The range of motion of the knee joint was set at 0–90°. The test protocol consisted of three repetitions at 60°.s^−1^ and five repetitions at 180°.s^−1^. Sixty and 180°.s^−1^ were chosen as they are reasonable and comfortable test velocities that seems to meet the essential requirements of testing validity and the need for information about muscle performance at the functional range. Moreover, as the higher test velocities incorporate lower reliability, more repetitions were necessary to avoid the concerns regarding the reliability of the tests. So, more repetitions were considered for the higher test velocities in this study [16]. The isokinetic measures included PTBWR and average power.

The torque-time curves were transformed into the frequency domain with Discrete Fourier Transform toolbox of MALTAB (The Mathworks, Inc. USA). Maximum frequency values contained within the specific signal power were chosen. In the previous study by Giakas [17] the criterion of 95% was used and in the other study by Tsepis [14] 90%, 95% and 99% level of signal power were used. Along with these three levels of signal power, we introduce the power level of 70.7% (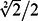) for further rang of signal content.

### *Statistical Analysis*

Data were entered into a statistical software package, SPSS 18(SPSS, Chicago, IL). As a consequence of the small sample size, we preferred to use the non-parametric tests [18]. The Wilcoxon signed rank test was used to compare the different test conditions for variables including: isokinetic tests, torque-time curve frequency contents, and functional tests. Furthermore, to compare ACLD with the normal group, Mann-Whitney U test was calculated. A p-value of 0.05 or less was considered significant. Spearman's Rank Correlation Coefficient was used to find any relationship between the peak torque generation capacity of the ACLD/healthy subjects and the FKB/sleeves effect. Brace effect was a new variable which was calculated as bellow for a better comparison of the braced and wearing sleeves conditions with the NBR condition in different variables [3], [9]:
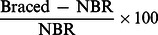



## Results

### Isokinetic Test

#### PTBWR

In the ACLD group in both isokinetic velocities (i.e.60 and 180°.s^−1^), wearing FKB/sleeve increased the extension peak torque for an average of 2.1% in 60°.s^−1^ and 4.7% in 180°.s^−1^. This effect was higher for wearing the FKB, more than 7.5%. Conversely, for normal subjects the FKB effect was negative. That means wearing the FKB has decreased the extension and flexion torques (respectively more than 3% and 10%), although none of the above differences were statistically significant (P>0.05). Peak torques comparisons showed no significant differences when they were compared between braced conditions. (Table 1)

**Table 1:** Brace Effect on Isokinetic Peak Torque.

Condition	Brace Effect for Peak Torque (%)^a^ ^b^							
	Normal				ACLD			
	Flexion		Extension		Flexion		Extension	
	60°.s^−1^	180°.s^−1^	60°.s^−1^	180°.s^−1^	60°.s^−1^	180°.s^−1^	60°.s^−1^	180°.s^−1^
Sleeve	0.36±13.7	5.71±11.6	−2.08±14.9	1.66±18.5	−11.56±16.3	0.08±10.9	−5.04±7.6	0.84±9.3
Sleeve with metal supports	−8.33±28.0	−5.20±22.3	−3.24±8.8	2.28±13.4	0.54±22.4	3.50±6.5	3.50±22.3	5.70±7.0
FKB^c^	−10.24±17.7	−10.31±14.0	−3.16±14.0	−3.77±13.9	−6.87±14.4	−0.28±20.6	7.85±12.9	7.65±10.6
Average	−6.07	−3.27	−2.83	0.06	−5.96	1.1	2.10	4.73
SD	5.6	8.2	0.6	3.3	6.1	2.1	6.6	3.5

a (braced - NBR)/NBR ×100.

b values are mean ± standard deviation.

c Functional Knee Brace.

Average Power: In the ACLD subjects the average power comparisons between the NBR and the FKB/Sleeve conditions revealed that in wearing both the FKB and neoprene sleeve with four metal supports increased the average power of the extension in 60 and 180°.s^−1^. There was a statistically significant increase in the extension average power measurement when wearing the FKB comparing to the NBR condition in 180°.s^−1^, z = −1.99, p = 0.046, with a large effect size (r = 0.57).

However, in the normal cases the FKB and neoprene sleeve with four metal supports reduced the average power in both flexion and extension, especially in 60°.s^−1^, although these effects were not statistically significant. (Table 2)

**Table 2:** Brace Effect on Isokinetic Average power.

Condition	Brace Effect for Average Power (%)^a^ ^b^							
	Normal				ACLD			
	Flexion		Extension		Flexion		Extension	
	60°.s^−1^	180°.s^−1^	60°.s^−1^	180°.s^−1^	60°.s^−1^	180°.s^−1^	60°.s^−1^	180°.s^−1^
Sleeve	2.04±18.3	7.37±18.5	−1.40±10.4	3.22±21.4	−7.67±15.8	1.90±23.5	−7.60±14.0	−3.01±15.7
Sleeve with metal supports	−9.00±31.2	−3.08±29.9	−5.53±11.7	2.66±17.0	−1.92±22.7	−4.22±13.0	1.01±20.7	10.48±10.8
FKB^c^	−7.78±21.4	−10.79±21.1	−6.41±11.4	−4.38±18.1	−6.22±17.7	4.23±43.8	5.54±12.1	12.58±13.6^d^
Average	−4.91	−2.17	−4.45	0.5	−5.27	0.64	−0.35	6.68
SD	6.0	9.1	2.7	4.2	3.0	4.4	6.7	8.5

a (braced - NBR)/NBR ×100.

b values are mean ± standard deviation.

c Functional Knee Brace.

d Significancy at P<0.05 level.

#### Correlation

**Figure 2:**
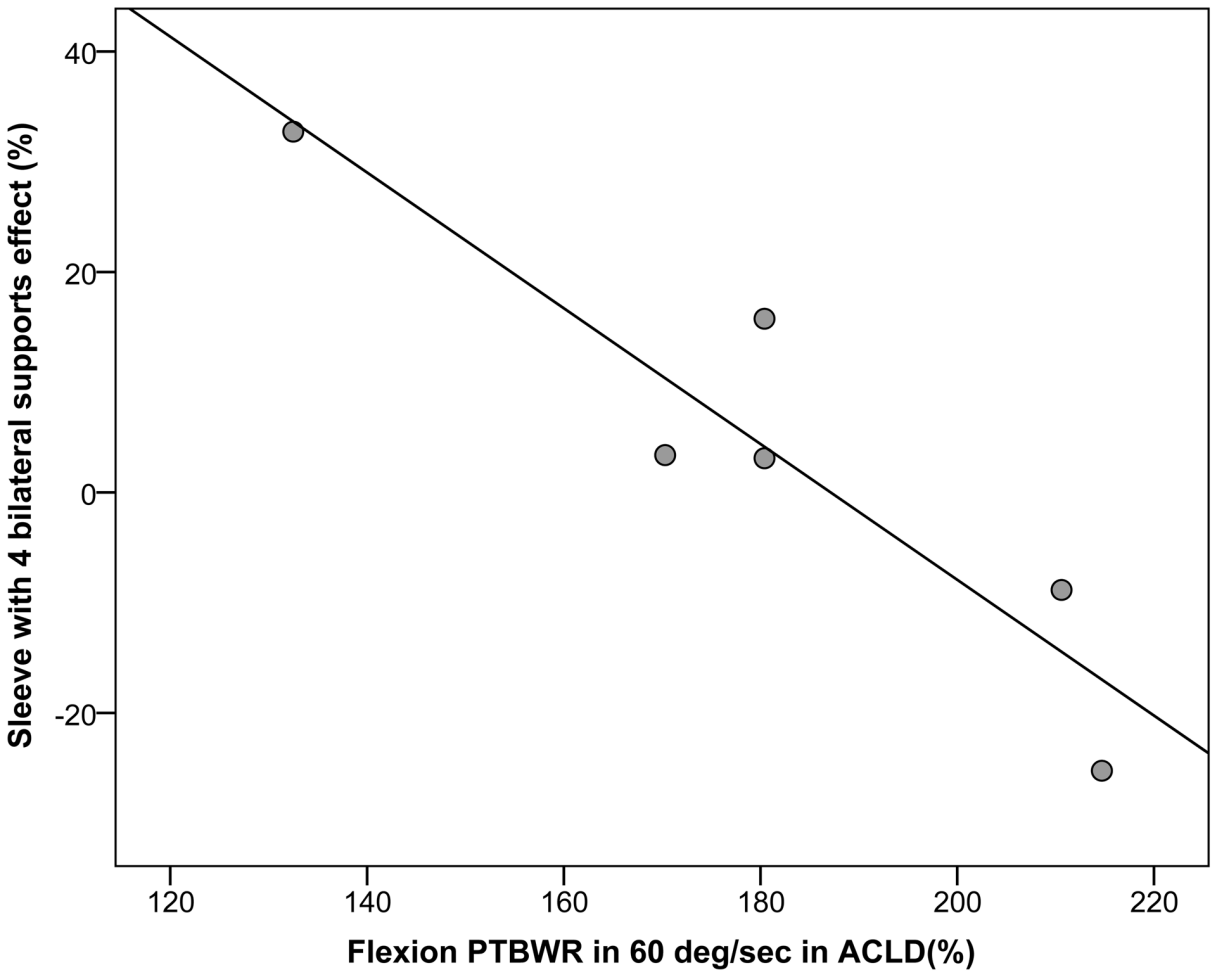
Correlation of the flexion PTBWR with sleeve with four metal supports effect, in ACLD subject at 60°.s^−1^; sleeve 2 in the figure legend denotes the one with the metal supports.

Results of correlation analyses revealed that there is some negative correlation between the extension PTBWR in NBR condition and the effect of neoprene sleeve with four metal supports in both normal and ACLD subjects. In ACLD subjects in 60°.s^−1^, there was a strong negative correlation between the PTBWR in NBR condition and the effect of sleeve with four metal supports in both flexion and extension (respectively rho = −1.00, p<0.001, and rho = −0.97, p = 0.017; Figure 2). In normal subjects this correlation was significant in 180°.s^−1^ in flexion (rho = −0.94, p = 0.005).

### Functional Tests

The results of the cross-over hop and SLVJ tests did not show any significant difference, comparing the three FKB/sleeves conditions with the NBR. However, as showed in Table 3, the FKB and sleeves adversely affected the hop test results in ACLD subjects (6.2%), while they had a small but positive effect on the normal subject (2.6%).

**Table 3:** Brace Effect on Functional Tests.

Condition	Cross-Over Hop (%)^a^ ^b^		SLVJ^c^ (%)^a^ ^b^	
	Normal	ACLD	Normal	ACLD
Sleeve	4.35±7.7	−5.13±4.9^d^	−0.22±4.0	−3.87±5.2
Sleeve with metal supports	1.67±9.2	−8.48±10.8	−4.49±6.1	−3.80±8.7
FKB^e^	1.65±8.9	−4.99±8.2	−1.19±4.1	0.85±8.7
Average	2.56	−6.2	−1.97	−2.27
SD	1.55	1.98	2.24	2.70

a (braced - NBR)/NBR ×100.

b values are mean ± standard deviation.

c Single Leg Vertical Jump.

d Significancy at P<0.05 level.

e Functional Knee Brace.

### Frequency content

In both ACLD and normal subjects, there have been no significant changes in the frequency content while they were wearing the FKB/sleeves comparing the NBR condition. However, as expanded in Table 4, in both groups, wearing the FKB/sleeves resulted in some increase in the frequency content in 60°.s^−1^, and caused a decrease in 180°.s^−1^.

**Table 4:** Brace Effect on Frequency Content.

Power Level (%)	ACLD^a^ ^b^						Normal^a^ ^b^					
	60°.s^−1^			180°.s^−1^			60°.s^−1^			180°.s^−1^		
	Sleeve	Sleeve with metal supports	FKB^c^	Sleeve	Sleeve with metal supports	FKB	Sleeve	Sleeve with metal supports	FKB	Sleeve	Sleeve with metal supports	FKB
70.7	31.09±67.3	45.45±58.6	17.52±16.5	12.24±22.0	−6.74±8.7	−1.69±10.2	51.54±98.0	24.04±53.6	46.36±94.7	0.93±18.0	−4.30±19.7	−0.37±16.9
90	1.35±20.6	9.19±19.7	1.52±7.7	12.89±21.0	−1.34±12.4	−1.86±11.9	22.64±44.9	13.13±26.0	21.10±41.9	−0.06±16.0	−0.74±11.5	−2.94±8.5
95	−1.11±11.70	6.47±6.1	5.43±3.7	2.60±7.6	−1.90±5.8	−2.61±5.1	10.25±20.6	7.49±11.3	9.18±17.9	−0.44±5.9	0.33±4.8	−1.53±3.4
99	−0.24±3.0	1.54±1.4	1.78±1.9	0.28±1.2	−0.48±1.9	−0.39±1.8	0.42±1.3	−0.14±1.7	0.44±2.1	−0.54±1.2	−0.13±1.4	−0.88±1.4

a (braced - NBR)/NBR ×100.

b values are mean ± standard deviation.

c Functional Knee Brace.

Moreover, the ACLD subjects had significantly higher frequency content at power level 70.7% in comparison to the normal group, mostly at 180°.s^−1^ of isokinetic velocity. (Table 5)

**Table 5:** Mann_Whitney Results for the Frequency Content Comparisons among ACLD subjects and normal group at power level 70.7%.

Normal/ACLD Comparison	Speed (°.s^−1^)	Z	r	P
NBR^a^	180	−1.92	0.56	0.054
Sleeve	180	−2.10	0.61	0.036
FKB^b^	180	−2.25	0.65	0.024

a Non-braced.

b Functional Knee Brace.

## Discussion

In the present study, a comprehensive analysis of the isokinetic performance of the knee muscles have been done in order to find the effect of wearing a FKB and two neoprene knee sleeves on the performance in ACLD and normal subject. The results revealed that the examined FKB and sleeves did not significantly improve or impaired the force control and force generation capacity of the knee joint in either ACLD or normal participants. However, wearing them had a weak positive effect on the isokinetic muscle performance of the ACLD group. Results of the SLVJ and crossover hop tests have also confirmed isokinetic findings; that is, wearing the FKB/sleeves did not have any effect on the performance of either tested groups.

**Figure 3:**
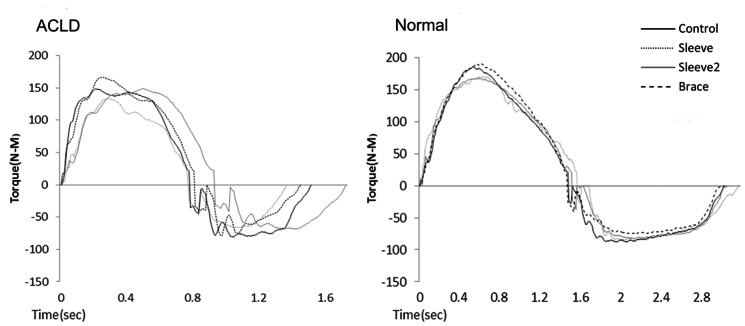
Torque-time curve in 4 conditions of test for extension and flexion at 180°.s^−1^ in an ACLD subject (left) and normal subject (right).

Isokinetic dynamometry can provide information about the different muscle groups' maximum capacity to generate torque in different angles throughout the range of joint motion in a controlled dynamic situation. Even small changes in hamstring strength are important because it functions as an agonist to the ACL and it can control anterior tibia transition especially in the absence of ACL [3], [19]. Also, the compressive stabilizing co-contraction of the two major knee muscle groups has a stabilizing effect for the ACLD knee [20]. Consequently, it is important to find out the changes that FKBs might impose on the knee muscles performance when they are used as rehabilitative and protective devices. Several studies have shown that when biologic signals are transferred from time domain to frequency domain, their rapid changes could be noted as high frequency contents [14], [17], [21] Therefore, Tsepis compared the frequency content of torque-time curve of concentric isokinetic knee flexion and extension in intact and ACLD knee of 30 ACL deficient subjects. The result showed significant higher oscillations and consequently less force control in the ACLD knee [13], [14]. This study suggested that utilizing the frequency content of the torque-time curve in the ACLD patients is a valid method in order to assess the isokinetic performance of these patients. Hence, the authors of the present study concluded that, besides studying the conventional isokinetic variables (peak torque and average power), using this method can provide us with a more precise comparison among the different braces, regarding their effect on the knee muscles performance. In the current study, in 180°.s^−1^ of isokinetic velocity, ACLD group showed considerably higher oscillations in the torque-time pattern comparing to the control group (p<0.05) (Figure 3, Table 5).The concurrence of these results with the previous study by Tsepis [14] shows that the methodology of the current one was appropriate. However, wearing none of the FKB/sleeves led to a smoother torque generation pattern in neither of the subjects groups.

There are few comparable studies using isokinetic dynamometry to examine brace effect on the knee muscle performance in ACLD patients [3], [10]. Wojtys examined the effects of six different FKBs on the isokinetic performance of five ACLdeficient patients at 60°.s^−1^. All of the braces reduced the amount of flexion and extension torques by averages of 5.8% and 2.4%, respectively. In the present study, the extension torques increased by an average of 2.1% for FKB and the sleeve with metal supports. In the flexion, FKB and the simple neoprene knee sleeve caused 6% torque reduction. In terms of average power measurements, results of Wojty's [3] study are consistent with the results of the present study; There was no significant change wearing most of the braces in extension (−0.4%), and in four of the braces the flexion power was decreased significantly (−12.3%) which is in agreement with the findings of the present study (the reduction of flexion power was about 5.3%, although this difference was not significant).

Also, Houston [10] reported that during isokinetic knee extension, wearing a hinged knee brace caused up to 30% reduction in torque output comparing to the NBR condition. One reason for the difference between the results of Houston study and the current one is that the patients were accustomed to wearing their braces. Moreover, subjects of the Houston's study had different complications including medial collateral ligament instability. Besides, brace designs were not the same.

In another study by Birmingham [9], isokinetic muscle performance of 27 ACL-reconstructed(ACLR) subjects were examined wearing a DonJoy Defiance brace in 90°.s^−1^ of angular velocity. Brace effect was significant only for flexion (−7.3%) which is in agreement with the results of our study for the FKB effect (−6.9%).

Lu et al. [22] conducted a study on the immediate effect of Donjoy Goldpoint brace on fifteen ACLD subjects through kinetic and kinematic measurements of the of the lower limb joint movements. ACLD subjects showed 6.3% greater extensor and 0.9% flexor knee moments with the knee brace in comparison with the unbraced condition, yet none of the differences were statistically significant which agrees with the findings of the present study; the FKB effect was 7.8% for the extension moment.

In the current study, the effect of FKB/sleeves has been also examined on the healthy subjects to eliminate all injury related biases. The FKB, neoprene knee sleeves and test protocol were exactly identical for the healthy and ACLD group. The brace effect on the peak torque and power in the control group revealed no significant effect for the FKB/sleeve comparing to the NBR although there have been insignificant reductions for both variables in extension and flexion (up to −6%). These results are in contradiction of the changes that the tested FKB and sleeves imposed on the ACLD subjects. This is consistent with the results of two other studies by DeVita and Tegner. Both of these studies had shown some negative effect in isokinetic muscle performance of the healthy athletes [23], [24]. Moreover, in another study by Mortaza et al. [12], similar isokinetic and functional tests were used to assess the effect of a prophylactic knee brace with a similar design on the performance of healthy collegiate football players. The result showed 3.2% and 0.4% moment reduction at 60°.s^−1^ for flexion and extension respectively, but in the healthy participants of the present study, wearing the FKB reduced flexion moment for 10.2% and extension moment for 3.2%. Although these brace effects were not statistically significant, the brace seemed to have a more negative effect on the healthy subjects comparing to the athletic subjects. These results confirm that the subjects' physical and strength status (e.g. athletic vs. non-athletic) could have an influence on the brace effect.

In addition, the correlation of subjects' muscle strength and FKB/sleeves effect was examined in ACLD and normal subjects. In both groups, mostly in ACLD group, there was a relationship between the effect sleeve with four metal supports and the peak torque generation capacity of the participants. That is, participants with less lower PTBWR have improved in generating peak torque when they were wearing the sleeve with four metal supports. This result is consistent with the previous studies, indicating that the effect of brace on the muscle performance could be depended on the strength and status of the ACL injured patients [9], [22], [25]. However, none of these isokinetic studies are on the ACLD patients.

Finding of this study also suggest that these braces could possibly be of benefit, depending on the current muscle strength and performance quality of the ACLD patients. And this must be considered when wearing a FKB is advised to ACLD patients. Moreover, in this study three different choices of knee support have been examined. It appears that wearing the knee sleeve with four metal supports and the FKB were beneficial to ACLD subjects although this effect was insignificant. Hence, considering that the FKB used in the present study had a metal frame and firm plastic shells, it may provide the ligaments of the knee with more support than the neoprene sleeve with metal supports. Moreover, in this study the simple neoprene sleeve was used to find out the possible restriction that the neoprene can impose on the performance as an underlying structure of a knee support; the results showed no significant restriction for neoprene sleeves specifically according to the isokinetic measurements in both ACLD and healthy groups. So, considering the positive effect of the neoprene sleeves on the proprioception of the knee particularly in the ACLD subjects [26], [27], using neoprene sleeves as a functional support for ACLD patients seems beneficial.

Hence, one of these knee supports could be chosen according to the level of risk that the ACLD patients might face (e.g. athletes).

One limitation of this study was that it was restricted to an assessment of the effects of bracing on the knee joint isokinetics and function. Further studies may also be helpful in investigating the interaction and compensation of adjacent joints to the knee (i.e. ankle and hip). This study was a preliminary study, so another one with a larger number of subjects may lead to more significant results.

## Conclusion

The results of the current study showed that the examined brace and sleeves did not have any negative or positive effect on the knee performance in either of the examined groups. However, the small positive effect of the brace/sleeves on the peak torque and average power in the ACLD group indicates that these brace/sleeves can reduce the bilateral asymmetry in ACLD patients. Moreover, use of these braces seems to be beneficial to this group of patients considering the protective effects of FKBs and the positive effects of neoprene sleeves on knee joint position sense in ACLD subjects.

## References

[1] Beynnon BD, Fleming BC, Churchill DL, Brown D (2003) The effect of anterior cruciate ligament deficiency and functional bracing on translation of the tibia relative to the femur during nonweightbearing and weightbearing. Am J Sports Med 31: 99–105. 12531765 10.1177/03635465030310012801

[2] Fleming BC, Renstrom PA, Beynnon BD, Engstrom B, Peura G (2000) The influence of functional knee bracing on the anterior cruciate ligament strain biomechanics in weightbearing and nonweightbearing knees. Am J Sports Med 28: 815–824. 11101104 10.1177/03635465000280060901

[3] Wojtys EM, Kothari SU, Huston LJ (1996) Anterior cruciate ligament functional brace use in sports. Am J Sports Med 24: 539–546. 8827316 10.1177/036354659602400421

[4] Cawley PW, France EP, Paulos LE (1991) The current state of functional knee bracing research. A review of the literature. Am J Sports Med 19: 226–233. 1867331 10.1177/036354659101900304

[5] Chew KTL, Lew HL, Date E, Fredericson M (2007) Current evidence and clinical applications of therapeutic knee braces. Am J of Phys Med Rhab 86: 678–686. 10.1097/PHM.0b013e318114e416 17667199

[6] Paluska SA, Douglas BM (2000) Knee Braces: Current Evidence and Clinical Recommendations for Their Use. American Family Physician 61: 411–423. 10670507

[7] Albright JP, Saterbak A, Stokes J (1995) Use of knee braces in sport. Current recommendations. Sports Med 20: 281–301. 8571003 10.2165/00007256-199520050-00001

[8] Rishiraj N, Taunton JE, Lloyd-Smith R, Woollard R, Regan W, et al (2009) The Potential Role of Prophylactic/Functional Knee Bracing in Preventing Knee Ligament Injury. Sports Med 39: 937–960. 19827861 10.2165/11317790-000000000-00000

[9] Birmingham TB, Kramer JF, Kirkley A (2002) Effect of a Functional Knee Brace on Knee Flexion and Extension Strength After Anterior Cruciate ligament Reconstruction. Arch Phys Med Rhab 83: 1472–1475. 10.1053/apmr.2002.35093 12370890

[10] Houston ME, Goemans PH (1982) Leg muscle performance of athletes with and without knee support. Arch Phys Med Rhab 63: 431–432. 7115043

[11] Veldhuizen JW, Koene FMM, Oostovogel HJM (1991) the Effects of a Supportive Knee Brace on Leg Performance in Healthy Subjects. Int J Sports Med 12: 577–581. 1797701 10.1055/s-2007-1024737

[12] Mortaza N, Ebrahimi I, Jamshidi AA, Abdollah V, Kamali M, et al (2012) The Effects of a Prophylactic Knee Brace and Two Neoprene Knee Sleeves on the Performance of Healthy Athletes: A Crossover Randomized Controlled Trial. PLoS ONE 7: e50110. 23185549 10.1371/journal.pone.0050110 PMC3503729

[13] Tracy BL, Enoka RM (2002) Older adults are less steady during submaximal isometric contractions with the knee extensor muscles. J Appl Physiol 92: 1004–1012. 11842033 10.1152/japplphysiol.00954.2001

[14] Tsepis E, Giakas G, Vagenas G, Georgoulis A (2004) Frequency content asymmetry of the isokinetic curve between ACL deficient and healthy knee. Clin Biomech 37: 857–864. 10.1016/j.jbiomech.2003.11.009 15111073

[15] Zachazewski JE, Magee DJ (1996) Return to Competition : Functional Rehabilitation. Athletic Injeries and Rehabilitation. Philadelphia: W.B. Saunders pp. 245–252.

[16] Devir Z (2004) Isokinetics of the Knee Muscles. In: Devir Z, editor. Isokinetics : Muscle Testing, Interpretation and Clinical Applications. 2nd ed. Edinburgh: Churchill Livingstone. pp. 150.

[17] Giakas G, Baltzopoulos V (1997) Time and frequency domain analysis of ground reaction forces during walking: An investigation of variability and symmetry. Gait Posture 5: 189–197.

[18] Seigal S, Castellan NJ (1988) Nonparametric Statistics for the Behavioural Sciences International Edition. New York: McGraw-Hill Book Co.

[19] Snyder-Macker L (2005) The Knee. In: Levangie PK, Norkin C, editors. Joint Structure and Function: A Comprehensive Analysis. 4th ed. Philadelphia: F. A. Davis Company. pp. 393–420.

[20] Myer GD, Ford KR, Barber Foss KD, Liu C, Nick TG, et al (2009) The relationship of hamstrings and quadriceps strength to anterior cruciate ligament injury in female athletes. Clin J Sport Med 19: 3–8. 19124976 10.1097/JSM.0b013e318190bddb PMC9928500

[21] Stergiou N, Giakas G, Byrne JB, Pomeroy V (2002) Frequency domain characteristics of ground reaction forces during walking of young and elderly females. Clin Biomech (Bristol, Avon) 17: 615–617. 10.1016/s0268-0033(02)00072-4 12243722

[22] Lu T-W, Lin H-C, Hsu H-C (2006) Influence of Functional Bracing on the Kinetics of the Anterior Cruciate Ligament-Injured Knees During Level Walking. Clin Biomech 21: 517–524. 10.1016/j.clinbiomech.2005.12.017 16494979

[23] DeVita P, Lassiter T, Hortobagyi T, Torry M (1998) Functional Knee Brace Effects During Walking in Patients With Anterior Cruciate Ligament Reconstruction. Am J Sports Med 26: 778–784. 9850778 10.1177/03635465980260060701

[24] Tegner Y, Pettersson G, Lysholm J, Gillquist J (1988) The effect of derotation braces on knee motion. Acta Orthop Scand 59: 284–287. 3381659 10.3109/17453678809149364

[25] Sforzo GA, Chen N-M (1989) The effect of prophylactic knee bracing on performance. Med Sci Sport Exerc 21: 254–257. 2733572

[26] Ramsey DK, Wretenberg PF, Lamontagne M, Nemeth G (2003) Electromyographic and biomechanic analysis of anterior cruciate ligament deficiency and functional knee bracing. Clin Biomech (Bristol, Avon) 18: 28–34. 10.1016/s0268-0033(02)00138-9 12527244

[27] Theoret D, Lamontagne M (2006) Study on three-dimensional kinematics and electromyography of ACL deficient knee participants wearing a functional knee brace during running. Knee Surg Sports Traumatol Arthrosc 14: 555–563. 16598506 10.1007/s00167-006-0072-3

